# Neighborhoods, Networks, and HIV Care Among Men Who Have Sex With Men: Proposal for a Longitudinal Study

**DOI:** 10.2196/64358

**Published:** 2024-11-13

**Authors:** Hong Van Tieu, Vijay Nandi, José E Diaz, Emily Greene, Melonie Walcott, Frank Curriero, Michael R Desjardins, Cara Wychgram, Carl Latkin, Andrew G Rundle, Victoria A Frye, NNHIV Study Team

**Affiliations:** 1 Laboratory of Infectious Disease Prevention Lindsley F. Kimball Research Institute New York Blood Center New York, NY United States; 2 Division of Infectious Diseases Department of Medicine Columbia University Irving Medical Center New York, NY United States; 3 Laboratory of Data Analytics Lindsley F. Kimball Research Institute New York Blood Center New York, NY United States; 4 Department of Medicine STAR Program SUNY Downstate Health Sciences University Brooklyn, NY United States; 5 City University of New York CUNY School of Medicine New York, NY United States; 6 Department of Health Policy, Management and Behavior College of Integrated Health Sciences State University of New York at Albany Albany, NY United States; 7 Spatial Science for Public Health Center and Department of Epidemiology Johns Hopkins Bloomberg School of Public Health Baltimore, MD United States; 8 Department of Health, Behavior and Society Johns Hopkins Bloomberg School of Public Health Baltimore, MD United States; 9 Department of Epidemiology Columbia University Mailman School of Public Health New York, NY United States; 10 Columbia University School of Social Work New York, NY United States

**Keywords:** neighborhoods, social networks, HIV care outcomes, men who have sex with men, living with HIV, activity spaces

## Abstract

**Background:**

The majority of people living with HIV in the United States are men who have sex with men (MSM), with race- and ethnicity-based disparities in HIV rates and care continuum. In order to uncover the neighborhood- and network-involved pathways that produce HIV care outcome disparities, systematic, theory-based investigation of the specific and intersecting neighborhood and social network characteristics that relate to the HIV care continuum must be engaged.

**Objective:**

Using socioecological and intersectional conceptual frameworks, we aim to identify individual-, neighborhood-, and network-level characteristics associated with HIV care continuum outcomes (viral suppression, retention in care, and antiretroviral adherence) among MSM living with HIV in New York City.

**Methods:**

In the longitudinal cohort study, we assess 3 neighborhoods of potential influence (residential, social, and health care access activity spaces) using Google Earth. We investigate the influence of neighborhood composition (eg, concentrated poverty and racial segregation) and four neighborhood-level characteristics domains: (1) community violence, physical disorder, and social disorganization (eg, crime rates and housing vacancy); (2) alcohol and other drug use; (3) social norms (eg, homophobia and HIV stigma); and (4) community resources (eg, social services and public transit access). We test theoretical pathways of influence, including stress or coping, stigma or resilience, and access to resources, across the different neighborhoods in which MSM live, socialize, and receive HIV care. At each visit, we locate each participant’s reported activity spaces (ie, neighborhoods of potential influence) and collect individual-level data on relevant covariates (including perceptions of or exposure to neighborhoods) and social network inventory data on the composition, social support, and perceived social norms. The outcomes are HIV viral suppression, retention in care, and antiretroviral adherence. These data are combined with an existing, extensive geospatial database of relevant area characteristics. Spatial analysis and multilevel modeling are used to test the main theory-driven hypotheses and capture independent neighborhood-level and network-level effects and changes over time.

**Results:**

The study began enrollment in March 2019 and concluded visits in December 2023, with a total of 327 participants enrolled. The median age was 44.1 (SD 11.5) years. Almost all participants self-identified as cisgender men (n=313, 98.1%) and as gay, homosexual, or bisexual (n=301, 94.4%). Overall, 192 (60.1%) participants identified as non-Hispanic Black, and 81 (25.3%) identified as Hispanic. Most (n=201, 63%) reported at least occasional difficulty in meeting basic needs (eg, rent and food) in the past 6 months. The mean number of years living with HIV was 15.4 (SD 10.1).

**Conclusions:**

This study will have direct implications for the design of multilevel interventions, addressing factors at the neighborhood, network, and individual levels. Results may inform urban planning and program design to improve HIV care outcomes for MSM, particularly for Black and Latino MSM living in urban areas.

**International Registered Report Identifier (IRRID):**

PRR1-10.2196/64358

## Introduction

In order to successfully implement treatment, as well as prevention, as a national strategy to end the HIV epidemic in the United States, it is crucial to address and bridge gaps in the HIV care continuum. The vast majority of people living with HIV in the United States are men who have sex with men (MSM). Among MSM, there are marked race- and ethnicity-based disparities in HIV infection rates and engagement and retention in HIV care. Compared with White MSM, Black and Hispanic or Latino (henceforth, Latino) MSM living with HIV are less likely to be on antiretroviral therapy (ART), adhere to ART, and achieve viral suppression [1-12]. Despite the long-standing focus on individual-level drivers, disparities in HIV acquisition and care engagement and retention among Black and Latino MSM are influenced by factors that are often beyond the individual such as the social determinants of health (SDH) [13]. The SDH can shape environments and behaviors that increase vulnerability to HIV acquisition and negatively affect HIV outcomes (eg, viral suppression, morbidity, and mortality rates). Yet the role of the SDH on HIV outcomes is not widely studied; thus, the mechanisms of action are not well understood.

Research on neighborhood factors and the care continuum suggests that poverty and racial segregation, as well as access to transit and distance to pharmacies and medical care, are associated with HIV care–related outcomes [14-19]. Similarly, social network-level factors, such as high levels of social support and HIV serostatus disclosure to social network members, are associated with improved HIV care outcomes [15,20-25]. Network and neighborhood characteristics are reciprocally related, with age composition, concentrated poverty, and racial segregation differentially fostering advantages and disadvantages for networks and residents alongside individual-level characteristics, such as socioeconomic status [26-28]. Still, additional research is needed to elucidate the pathways through which networks and neighborhoods foster racial and ethnic disparities in HIV care outcomes. The results of such analyses will provide an empirical basis for interventions and policies to fill gaps in the care continuum among Black and Latino MSM. To reach that goal, a systematic, theory-based investigation of the specific and intersecting neighborhood and network characteristics that relate to retention in care, ART adherence, and viral suppression must be used.

In this longitudinal study, which was recently completed with data analysis ongoing, we enrolled MSM living with HIV in New York City to complete study visits every 6 months, up to either 12 (post–COVID-19 cohort) or 36 (pre–COVID-19 cohort) months, to determine whether and how neighborhood and network characteristics influence their HIV care. Using socioecological and intersectional stigma and discrimination conceptual frameworks, we aim to examine neighborhood- and network-level characteristics that influence different aspects of the HIV care continuum among MSM living with HIV in New York City in a longitudinal study. We focus on 3 reported activity spaces (ie, potential neighborhoods of influence)—where MSM reside (home neighborhood), where they socialize and spend the most time (social neighborhood), and where they access their HIV medical care (health facility access neighborhood). We investigate the influence of four neighborhood-level characteristic domains: (1) community violence, physical disorder, and social disorganization (eg, interpersonal crime rates, concentrated poverty, and racial segregation); (2) neighborhood alcohol and other drug (AOD) use-associated factors (eg, alcohol outlets); (3) neighborhood-level social norms (eg, homophobia, HIV stigma, racial discrimination, and health norms); and (4) community resources (eg, HIV prevention services, social services, public transit access, and greenspace) and how these play out in the different neighborhoods in which MSM live, socialize, and receive HIV care.

## Methods

### Study Design

The Neighborhoods, Networks and HIV Care among Men Who Have Sex with Men (NNHIV) study is a longitudinal cohort study that examines neighborhood and social network factors that influence HIV care outcomes (retention in care, ART adherence, and viral suppression) using spatial analysis and multilevel modeling among MSM living with HIV in New York City. The study investigates the influence of neighborhood composition and 4 neighborhood-level domains, testing theoretical pathways of influence across the different neighborhoods in which MSM live, socialize, and receive HIV care.

### Ethical Considerations

This study has been reviewed and approved by the New York Blood Center institutional review board (1066143). Written informed consent was obtained for participants 18 years of age and older who took part in the longitudinal study; an assent process was in place for any participants aged 16-17 years along with the use of a minor advocate. Participants received US $100 for their time and travel at the baseline visit and at each follow-up visit.

### Eligibility Criteria

Eligibility criteria included (1) reporting male sex at birth, (2) self-report being HIV-positive (including being newly diagnosed with acute HIV infection), (3) engaging in anal sex with a man in the past 6 months, (4) at least 16 years of age, (5) reside within the 5 New York City boroughs, (6) able to read and respond in English, and (7) provide written informed consent (or assent if between 16 and 18 years of age, not inclusive of 18 years). Only cisgender men were included in this study. We excluded other populations, including cisgender and transgender women, because the study specifically seeks to understand the influence of neighborhoods and networks on HIV care outcomes among MSM living with HIV, and therefore, would not be optimally designed to capture the unique needs and barriers impacting women.

### Sample Size

Sample size, power, and effect size considerations focused on longitudinal logistic regression models with neighborhood-, network-, and individual-level variables included as covariates. We aimed to enroll at least 40% who self-identify as Black, non-Hispanic and 30% who self-identify as Hispanic or Latino. Effect size calculations were performed assuming a minimum of 40% (n=220) of Black, non-Hispanic MSM and 30% (n=165) Latino MSM. Fixing type I error rate at 0.05, a sample size of 550 MSM, within-person correlation of 0.5, statistical power of at least 80%, and 10% attrition (loss to follow-up), minimum effect sizes (in terms of odds ratios) for detecting any difference in neighborhood and network effects on HIV care outcomes over the 5-visit, 24-month period range from 1.14 to 1.23. The proposed sample size of 550 MSM was later modified to 327 given the challenges of recruitment experienced during the COVID-19 pandemic.

### Recruitment

We enrolled a total of 327 MSM. Initially, convenience sampling for recruitment was used to recruit participants, which included in-person recruitment at venues including bars clubs, gay pride events, and HIV clinics and service organizations, internet-based ad placements (eg, Craigslist [Craigslist Inc], geospatial dating applications such as Grindr [Grindr Inc]), and referrals from enrolled participants (maximum 3 referrals per participant). Later, the recruitment strategy was revised to use a modified, venue-based, time-space sampling methodology, to ensure a diverse sample to increase generalizability. For in-person and internet-based recruitment, the sampling frame of venues included a range of neighborhoods across the 5 New York City boroughs (from traditionally considered gay enclaves to those with a less visible gay presence, from places where known HIV-infected individuals frequent to lesser-known areas) and a range of venues within neighborhoods, such as bars, clubs, HIV clinics and service organizations, gay pride events, and street locations. Each month, a sample of 8 to 16 locations and day-time periods were randomly selected.

Participants who enrolled and completed a study visit were provided with referral cards (either in person, via email, or text) in which they could refer their friends to the study, a maximum 3 referrals for US $10 for each referral who successfully screened for the study and enrolled into the study (maximum US $30 for total 3 referrals); this referral system was later amended with a limit of 1 referral per participant to reduce referral bias. For internet and mobile app–based recruitment, banner ads were placed on select websites and social media apps, including Facebook (Meta Platforms), Craigslist, Instagram (Meta Platforms), and Grindr.

Participants were asked to complete a brief internet-based eligibility screener if they indicated interest in participating in the study. Potentially eligible (based on the results of the screener) participants were asked to provide contact information to schedule their study visit. At the time their study visit appointment was scheduled, the participant was asked to bring in a copy of their prior HIV diagnosis report to the visit, if available.

Study enrollment began in March 2019 and was paused in March 2020 with an enrollment of 213 participants prior to the COVID-19 pandemic lockdown in New York City (henceforth “pre-lockdown cohort”). As a result, recruitment and enrollment into the study was paused from March 2020 to August 2021. Follow-up visits for the 213 participants in the pre-lockdown cohort used a hybrid visit model initially during the beginning of the pandemic (remote and in-person procedures), followed by a transition back to all in-person visits; follow-up visits were extended from month 24 to month 36 for a longer follow-up period for the pre-lockdown cohort. The study reopened for enrollment in August 2021 with 114 participants enrolled (“post-lockdown cohort”) before enrollment finally closed in November 2022. The post-lockdown cohort participants were followed to month 12, with the last visits completed in December 2023. A total of 327 participants enrolled in the longitudinal study.

### Study Visit Procedures

#### Baseline Visit Study Procedures

Study visits took place at our research site in Manhattan. After written informed consent (assent for those aged 16-17 years), participants underwent a rapid HIV antibody test (Orasure Oraquick ADVANCE Rapid HIV-1/2 Antibody) to confirm their self-reported HIV status if they did not bring a copy of their prior HIV diagnosis report. Participants were also asked to bring their ART medications for a pill count by staff and to sign a medical release form, with medical provider office visit notes obtained to confirm attendance and missed visits. They also had blood collection, which was sent to a local laboratory for HIV viral load and CD4 T lymphocyte (CD4 T cell) count testing. Test results were provided to participants, and referrals for HIV medical care and risk reduction counseling were provided and documented, as needed. The study visit ended for those who did not provide an HIV status report or whose HIV status was not confirmed.

Participants were then asked to identify their home, social, and health facility access neighborhoods by a trained research counselor using a neighborhood locator questionnaire and a desktop version of Google Earth. First, the interviewer asked for the name of their home neighborhood based on a drop-down menu of all 343 New York City neighborhoods. The interviewer then assisted participants in using Google Earth to “drop a pin” at the closest intersection near their home (home neighborhood), with the latitude and longitude coordinates of the pin drop recorded. Data on the perceived activity spaces (geographical neighborhoods) were collected with the question “When you think about your home neighborhood, what area do you usually think of?” with response categories “the block you live on, the area within (5 blocks/10 blocks/larger than 10 blocks) around the place you live.” This process was repeated for the neighborhood where they most often hang out or spend the most time (social neighborhood), and where they most often went for their HIV medical care in the prior 6 months, as well as the pharmacy where they get your HIV medications filled in the last 6 months (health facility access neighborhoods).

Participants then completed an egocentric social network inventory (SNI) which provides network-level covariate data. The inventory was administered by a trained research counselor, who entered participant responses into a computer-based program (EgoWeb 2.0 for Windows). For the social network inventory, participants were asked to name persons, using a name generator, whom they could rely on for support in the domains of (1) companionship—who do you get together with to spend time talking, relaxing, or hanging out with?; (2) confidant—who do you talk to about very personal and private things?; (3) financial—who would you ask to lend you US $100 if you needed it?; (4) housing—who would provide you with housing or a place to stay a few days or more if you needed it?; (5) medical—who would go to a medical appointment, such as to an HIV provider or mental health provider, with you?; and (6) medication adherence—who encourages you to take your HIV medications regularly or who you could talk to about problems with taking your HIV medications? In order to construct a comprehensive network composition of the participants, there were no restrictions on the number of network members named. The attributes that were asked about each social network member are (1) demographics (eg, age, gender, and race or ethnicity); (2) social member relationship type (sex partner, friend, family member, coworker, etc); (3) HIV status and HIV serostatus disclosure; (4) home borough and proximity (live within 20 blocks of ego); (5) communication, including duration of relationship, frequency and content of communication, distance, conflicts, and support; (6) perceived behavioral and attitudinal norms including norms for AOD use, ART adherence, medical care follow-up, HIV serostatus disclosure, and medical mistrust; (7) whether ART drugs are shared or sold to or bought from network member; and (8) whether the person knows other members of the social network. In addition, the participants provided information about social support from each named social network member, adapted from the Inventory of Socially Supportive Behaviors [29], consisting of 19 items grouped into 3 sub-domains to assess cognitive guidance (9 items, eg, [network member] suggested some action that you should take), emotional support (5 items, eg, [network member] expressed interest and concern in your well-being), and tangible assistance (4 items, eg, [network member] gave you over US $50).

Next, participants completed the computer-assisted survey instrument (CASI) questionnaire, with the first part of the CASI involving ART use and pill count completed with study staff and then the rest of the CASI completed alone in private with staff assisting as needed. The CASI included measures that tap into common (eg, depression, anxiety, and substance use perceived patient-provider interaction) and network and neighborhood-level factors that may also be related to HIV care outcomes (eg, perceived racism in health care, homophobia and HIV stigma, empowerment, and coping, racial or ethnic and gay community attachment and acculturation; Table 1).

**Table 1:** Independent variables.

Measure			Variables (measures)
**Sociodemographics**			
	Demographics	Age, socioeconomic status (SES; income, education, and employment housing or food insecurity), housing status, public assistance, place of birth, primary and secondary languages, marital or live-in status, current living situation, and arrest or incarceration history	
**Health status or health care**			
	Medical history	Year of HIV diagnosis, transmission risk, Centers for Disease Control and Prevention (CDC) stage of HIV disease, non-HIV medical comorbidities, ART^a^ adherence self-efficacy (refers to a person’s confidence to carry out behaviors related to adhering to treatment plans, HIV Treatment Adherence Self-Efficacy Scale [HIV-ASES] [30]), adherence motivation [31], perception of quality of HIV care [32], health literacy [33,34]. Availability of and access to ART adherence support, HIV care coordination or medical case management, and outreach programs with health care facility or provider [35,36], availability of and access to pillboxes or reminders or calendars from pharmacy or HIV medical provider [37]. History of diversion of ART drugs, and if so, in which neighborhoods [38-40]. Medical mistrust based on the HIV Conspiracy Beliefs Scale [41,42]	
	Health care and perceived racism in health care	Health insurance coverage and type of care, usual place of care, access to HIV care [43], number and type of HIV medical providers, reasons for change in providers, type of HIV medical care setting (academic, clinic, community-based organization, etc), and access to and usual pharmacy (retail vs independent vs mail order). Perceived racist treatment from health care providers (doctors’ offices and hospitals): 4-item instrument [44,45]	
	Perceived patient-provider interaction	Perceived quality of patient-provider interaction is based on 15 items from reactions assessments on the following domains: perceived quality of information, perceived level of difficulties of communication, and perceived affection and respect [46,47]	
**Sexual history**			
	Sexual behaviors	Number of male, female, and transgender partners in the last 6 months, partner type (primary, casual, etc), partner concurrency, frequency of vaginal, anal sex with, or without condoms and with or without alcohol and other drug use in the last 6 months, HIV serostatus of partner, and HIV serostatus disclosure to or by partner	
**Substance use**			
	Alcohol and drug use	Alcohol, Smoking, and Substance Involvement Screening Test (ASSIST) [48], adapted for 6 months intervals	
**Psychological and psychosocial**			
	Psychological distress and depression	Depression: Patient Health Questionnaire (PHQ-9) [49], anxiety: Generalized Anxiety Disorder (GAD-7), how often in last 30 days they felt nervous, restless or fidgety, worthless [50], occurrence of traumatic life events and post-traumatic stress disorder [51], and stressful life events in last 6 months [50]	
	Racial discrimination and stigma	Social stress and social discrimination (every day unfair treatment) [50], schedule of racist events: an 18-item scale that measures subjective experiences of racism across multiple domains (employment, education, housing, etc) [52]	
	Homophobia and HIV stigma	Experiences of homophobia: scale capturing experiences with homophobia as children and adults [53]; Internalized homophobia scale [54] based on (1) public identification about being gay, (2) stigma associated with being considered unattractive in the gay world and stigma associated with being gay outside the gay world, (3) social comfort with other gay men, and (4) moral and religious acceptability of being gay. Internalized HIV stigma [55], anticipated HIV stigma from friends or family, and neighbors or community and health care workers [56]	
	Empowerment	Adaptation of Rogers and colleagues’ scale [57] assessing empowerment among mental health consumers	
	Fatalism	External locus of control of health (God Locus of Health Control Scale (GLHC) scale) [58], 6-item scale that measures belief that God is in control of health and health conditions (eg, “God is directly responsible for my HIV getting better or worse”)	
	Machismo	Traditional Machismo and Caballerismo Scale [59,60]	
	Coping	Brief Coping Orientation to Problems Experienced Inventory (brief-COPE) [61]: 28-item measure of positive and negative (avoidant) coping responses to life problems	
	Racial or ethnic and gay community attachment and acculturation	Multigroup Ethnic identity Scale: 21-item scale that measures 4 sub-domains: positive ethnic attitudes and sense of belonging, ethnic identity of achievement, ethnic behaviors and practices, and other-group orientation 5-item scale assessing attachment to “a gay community” [62]; 12-item scale assessing attachment to the gay community in NYC^b^ [63]; 5-item scale assessing attachment to “a gay community” [62]; 12-item scale assessing attachment to the gay community in NYC [63]; and acculturation [64]	

^a^ART: antiretroviral therapy.

^b^NYC: New York City.

#### Follow-Up Study Visit Procedures

Every 6 months for up to 12 months (post–COVID-19 lockdown cohort, after August 2021) or 36 months (pre–COVID-19 lockdown cohort with participants enrolled prior to the onset of the COVID-19 pandemic in New York City in March 2020, which caused the city to go on pause or lockdown), participants completed the neighborhood locator questionnaire using Google Earth, SNI, and CASI questionnaire similar to those completed at the baseline visit; they underwent CD4 T cell and HIV viral load testing that were sent to a local laboratory. To assess network turnover, the SNI collected information on any new social network members from the prior 6 months and on any persons named previously who were no longer part of the networks. Medical records review and pill count were performed. Participants were reimbursed US $100 at each follow-up visit.

If participants were unable to come to the study site for their follow-up visit (for various reasons, including during lockdown in New York City during the COVID-19 pandemic), they were provided with the option of completing part of the follow-up visit remotely. For this remote visit, they were contacted by staff by secure videoconference or phone and completed surveys remotely on a computer via secure links. Participants then made an appointment with study staff to come into the site in person within 2 weeks for HIV viral load and CD4 T cell count testing.

### Study Retention

At the baseline visit, extensive locator information was obtained using a locator form, including the participant’s address and phone number. We also collected names, telephone numbers, and addresses of at least 2 secondary contacts, with an emphasis on stable relatives both within and outside of New York City, which allowed us to maintain contact with residentially mobile or marginally housed individuals. We asked the participant for specific instructions on how to leave messages to protect confidentiality. Retention efforts for the longitudinal study included reminders in between study visits, use of contacts, telephone reminders prior to follow-up visits, texts, and emails. If a participant missed a visit, the retention protocol was initiated by study staff, which included letters sent to participants’ homes and reaching out to the listed secondary contacts on the locator form.

### Statistical Analysis

#### Measures

The primary biological outcome is viral suppression (HIV viral load <20 copies per mL based on testing). Secondary outcome measures include retention in care (ie, >1 visit with an HIV medical provider in the last year) and retention in continuous care (ie, >2 visits at least 3 months apart with an HIV medical provider in the last year) as reported by CASI and confirmed by medical records review. ART adherence is based on the AIDS Clinical Trials Group (ACTG) [31] 4-question adherence instrument as reported by CASI and confirmed by pill count. Participants are categorized as adherent if they report taking ≥85% of the dispensed dosage. The geospatial database, which characterizes the demographic and socioeconomic conditions of the neighborhoods, provides data for the four neighborhood characteristic domains of interest that are (1) community violence, physical disorder, and social disorganization; (2) neighborhood AOD use-associated factors; (3) neighborhood-level social norms; and (4) community resources.

The geospatial database, which characterizes the demographic and socioeconomic conditions of the neighborhoods, provides data for the four neighborhood characteristic domains of interest that are (1) community violence, physical disorder, and social disorganization; (2) neighborhood AOD use-associated factors; (3) neighborhood-level social norms; and (4) community resources.

Table 1 lists the independent variables that are collected by CASI. Data on the participants’ perceptions of neighborhoods were obtained by CASI (Table 2) while Table 3 lists neighborhood characteristics based on archival data. As there are no area-level indicators available for some measures, select constructs from Table 2 will be aggregated up to the area level to create neighborhood-level variables used in the analyses, similar to prior work [65-67].

**Table 2:** Neighborhood characteristics assessed by participants.

Measure	Definitions
Exposure to neighborhoods	Level of exposure to both residential and other neighborhoods, including length of residence, who lives with them, assigned housing, how much time they spend in their neighborhoods, and how much time they spend in other neighborhoods [68,69]
Social ties	Number of friends or relatives respondents have in the neighborhood and the frequency with which the respondents see friends and relatives based on Sampson’s assessment of social ties [70]
Social cohesion and trust	Perceptions of connectedness and trust in their community [71-73]
Social or neighborhood involvement	Degree to which respondent is involved in local community or other groups (eg, local neighborhood groups or block associations), degree to which respondents interact with their neighbors will be assessed, using a sociability measure [71]
Perceived disorder, safety, and community violence	Perceived social disorder and physical decay based on Ross and Mirowsky [74]; Screen for Adolescent Violence Exposure (SAVE): 32-item self-report measure to assess frequency and type of violence [75]
Neighborhood integration, sense of community, and identification with neighborhood	Neighborhood integration will be measured using Perkin’s Sense of Community Scale [76]. 12-item 3D Strength of Identification Scale assesses self-reported feeling of identification with residential neighborhood [77]
Drug market presence	Presence of open air drug markets, street corner sales, corner store or bodega sales and known residence-based sales will be assessed directly using adaptation of previous assessments [78]
Social norms: drug use, sexual behaviors, violence, HIV medical care, and general health	Questions modified from the National Survey on Drug Use and Health [79] to measure behavioral and attitudinal norms around drug use and violence. Perceptions of neighborhood social norms concerning multiple partners, safer sexual behaviors, condom use, receiving HIV medical care, and general health
Social norms: neighborhood homophobia and HIV stigma or discrimination	Brief 6-item scale assesses experiences of and responses to race and sexual orientation–based discrimination in different domains [80], HIV stigma will be assessed using Earnshaw’s HIV stigma framework [56]

**Table 3:** Neighborhood characteristics from archival data.

Dimension		Data
**Community violence, physical disorder, and social disorganization**		
	Crime or social disorder	Crime risk index (overall crime, personal crime, murder, rape, robbery, assault, property crime, burglary, larceny, and motor vehicle theft); acceptably clean streets and sidewalks; and domestic and gender-based violence and hate crimes
	Built environment	Housing vacancy and Area Deprivation Index (ADI)
	Sociodemographics	Sex ratio, race or ethnicity, immigration status, linguistic isolation (families in which all members ≥14 years have at least some difficulty speaking English) [81-83], poverty, unemployed, on public assistance, proportion living in the same house for the previous 5 years, proportion of owner-occupiers, and same sex household
**Neighborhood AOD^a^ use-related factors**		
	Alcohol outlets	Alcohol outlets (National Establishment Time Series [NETS] [84] database)
**Community resources**		
	Community-based organizations, food pantries, and prevention services	InfoShare listing of all nonprofits, services, and public assistance
	Health care facilities and pharmacies	NETS database [85] include hospitals, health clinics, community-based care facilities, and so forth; pharmacies or drug stores
	Public transport access	Minimum distance to subway or bus and fewer bus stops
	Parks	Percent of tract covered by park and park cleanliness
**HIV-related data**		
	HIV prevalence, incidence, and related data	HIV prevalence, new diagnoses, pre-exposure prophylaxis (PrEP), testing, linkage to care, late diagnoses, receipt of care, viral suppression, and mortality [86]

^a^AOD: alcohol and other drug.

#### Data Analysis 1: Clustering and Spatial Trends in HIV Care Outcomes

First, we will use spatial analysis to characterize clustering and spatial trends in HIV care outcomes (retention in care, ART adherence, and viral suppression) among MSM living with HIV in the study. Mapping the data distributions of outcomes will provide visual appraisals and display potential spatial variations. We will measure and compare distances between home, social, and health facility access locations and the extent to which there is neighborhood overlapping. We will quantify patterns and relationships describing spatial variation at both the smaller scale (spatial clustering) and larger scale (spatial trends), and examine the association of sociodemographic covariates such as race or ethnicity, socioeconomic status, and neighborhood exposure with spatial clustering of the HIV care outcomes. Results will provide important insights into how social and spatial environments impact the outcomes and help inform our other regression analyses by providing baseline information on trends and variability that will be incorporated in the covariates in the regression models, both at the individual and neighborhood levels.

#### Data Analysis 2: Baseline Associations Between Exposure to Neighborhoods and HIV Care Outcomes

Second, we will conduct baseline analyses to assess associations between exposures to neighborhoods of potential influence (home, social, and health facility access) separately for each of the HIV care outcomes using logistic regression models. Univariate logistic regressions will be computed for the full set of independent variables describing the neighborhood of potential influence to assess the effect and contribution of each variable separately. Variables significant at the *P*<.10 level will be considered for multivariate analysis and longitudinal analyses to examine HIV care outcomes by race or ethnicity among MSM living with HIV in relationship to longitudinal changes in (1) exposure to neighborhoods of potential influence (home, social, and health facility access); (2) neighborhood characteristics in four conceptual domains (community violence, physical disorder, or social disorganization; neighborhood AOD use-associated factors; neighborhood-level social norms; and community resources); and (3) social networks.

#### Data Analysis 3: Associations in Changes Over Time Exposure to Neighborhoods and Social Networks and HIV Care Outcomes

We will use multilevel modeling to assess associations among changes over time in exposure to the 4 neighborhood characteristic domains and changes in social networks with HIV care outcomes.

## Results

The study was initially funded in August 2017, with additional multiyear funding in April 2019. The study began enrollment in March 2019 and concluded visits in December 2023, with a total of 327 participants enrolled (213 in the pre–COVID-19 pandemic lockdown cohort and 114 in the post–COVID-19 pandemic lockdown cohort). The study paused enrollment from March 2020 to August 2021 as a result of the COVID-19 pandemic, with the resumption of enrollment in August 2021 to November 2022. The participants in the prelockdown cohort completed study visits up to 36 months, while those in the postlockdown cohort completed visits up to 12 months. Results are planned for publication in the spring of 2025.

Baseline sociodemographic characteristics for 319 participants with available data are described in Table 4, with 205 participants in the prelockdown cohort and 114 in the postlockdown cohort. The median age was 44.1 (SD 11.5) years. Almost all the participants self-identified as cisgender men (n=313, 98.1%) and as gay, homosexual, or bisexual (n=301, 94.4%). Overall, 192 (60.1%) participants identified as non-Hispanic Black, 81 (25.4%) as Hispanic, 32 (10.0%) as non-Hispanic White, and 14 (4.4%) as other. More than half had any college education (n=175, 54.8%), while 216 (67.7%) reported an annual household income of US $20,000 or less and 280 (87.8%) had Medicare, Medicaid, or New York State AIDS Drug Assistance Program (ADAP) insurance coverage. Most (n=201, 63%) reported at least occasional difficulty in meeting basic needs (eg, rent and food) in the past 6 months. The mean number of years living with HIV was 15.4 (SD 10.1).

**Table 4:** Baseline sociodemographic characteristics of NNHIV^a^ study cohort.

		Total (N=319)	Prelockdown cohort (n=205)	Postlockdown cohort (n=114)
**Age (years), mean (SD)**		44.1 (11.5)	43.5 (11.6)	45.2 (11.4)
**Male, n (%)**		313 (98.1)	205 (100.0)	108 (94.7)
**Gender identity, n (%)**				
	Straight or heterosexual	3 (0.9)	3 (1.5)	0 (0.0)
	Gay, homosexual, or bisexual	301 (94.4)	192 (93.7)	109 (95.6)
	Other	15 (4.7)	10 (4.8)	5 (4.4)
**Race or ethnicity, n (%)**				
	Non-Hispanic Black	192 (60.2)	140 (68.3)	52 (45.6)
	Hispanic	81 (25.4)	44 (21.5)	37 (32.5)
	Non-Hispanic White	32 (10.0)	12 (5.9)	20 (17.5)
	Other	14 (4.4)	9 (4.4)	5 (4.4)
**Education level, n (%)**				
	No high school diploma	58 (18.2)	50 (24.4)	8 (7.0)
	Completed high school	86 (27.0)	59 (28.8)	27 (23.7)
	Some college or 2-year degree	103 (32.3)	64 (31.2)	39 (34.2)
	College or higher	72 (22.6)	32 (15.6)	40 (35.1)
	Annual personal income of US $20,000 or less	216 (67.7)	170 (82.9)	46 (40.4)
**Health insurance, n (%)**				
	Private	24 (7.5)	6 (2.9)	18 (15.8)
	Medicare, Medicaid, or ADAP^b^	280 (87.8)	189 (92.2)	91 (79.8)
	Do not know or prefer not to answer	15 (4.7)	10 (4.9)	5 (4.4)
	Unstable housing	29 (9.1)	24 (11.7)	5 (7.0)
	Not enough money for basic needs (eg, rent and food) in the past 6 months	201 (63.0)	140 (68.3)	61 (53.5)
**Years since HIV diagnosis**				
	Mean (SD)	15.4 (10.1)	15.3 (10.2)	15.8 (9.9)
	Value, n	298	192	106

^a^NNHIV: Neighborhoods, Networks and HIV Care among Men Who Have Sex with Men.

^b^ADAP: AIDS Drug Assistance Program.

## Discussion

### Principal Findings

Significant racial or ethnic disparities in HIV care–related outcomes among MSM in the United States motivate us to understand whether and how neighborhood and network characteristics act and interact to influence these outcomes. This study has several innovative features. First, neighborhoods and networks are rarely static and the HIV care continuum is a dynamic process in which individuals with HIV move on and off steps of the continuum. Given the limited data available on neighborhood and network dynamics among MSM living with HIV, this study’s longitudinal design examines changes in exposure to neighborhoods and social networks over time and determines how these influence HIV care outcomes, elucidating the potentially unique mechanisms by which they impact the HIV care continuum for MSM living with HIV by race or ethnicity. This design positions the study team to capture differential exposure to neighborhood and network characteristics and effects of national health policy changes, including changes to local, state, and federal health and social policy that may occur over time.

Second, we focus on identifying how individual-level factors mediate the impact of neighborhood-level factors, enabling us both to identify malleable characteristics that are amenable to traditional individual-level intervention and area-level factors, which, if modified, may have important positive effects on a range of health outcomes among MSM living with HIV. In addition, we investigate multiple neighborhood contexts for MSM living with HIV in the urban environment, rather than only their residential neighborhoods, which has been the focus (and a key limitation) of the majority of neighborhood research. We use innovative spatial analysis methods that will address limitations inherent in prior neighborhood-level research. We use Google Earth to identify the location and scale of multiple neighborhoods that are potentially important to the MSM. We collect neighborhood boundaries that are defined and characterized by the participants, which will reflect their own local, small-area definitions of the size of influential space in these contexts. Furthermore, the incorporation of subjective (perceptions) and objective measures of neighborhoods is innovative and has been not used in prior research examining the disparity in HIV care outcomes among MSM.

One area of focus of our analysis is HIV stigma, which has been associated with significant negative health and social outcomes among people with HIV [87,88]. HIV stigma influences the HIV care cascade by reducing access to testing [89,90] and medical treatment [91,92]. It also reduces the uptake of biomedical prevention technologies, critical tools to interrupt HIV transmission [89-95]. There is limited data on how community-level HIV stigma influences HIV care-related outcomes. In this study, we will examine the impact of individual-level intersectional stigma (internalized, experienced, and perceived community) at various levels on HIV care outcomes and will evaluate these relations over time.

A second area of focus is on stress and coping. According to stress theory, stressful life events give rise to coping responses, which can be adaptive or maladaptive. When a coping response is adaptive and effective, the impact of the stressor is mitigated; when the response either does not exist or is maladaptive, the stressor has a negative effect on the individual [96,97]. Neighborhood environmental conditions, such as concentrated poverty, racial segregation, physical disorder, and violence, increase the likelihood of experiencing stressors. Physical disorders (eg, vacant buildings) can lead to stressful experiences, such as crimes and assault, as they provide spaces and cover for illegal activity (eg, drug trade) [98-102], increasing general arousal or anxiety, and consequent negative immune function. There is literature on community violence and adverse health outcomes [103-105]. We will examine the impact of neighborhood-level concentrated poverty, racial segregation, and physical disorder on stressful life events and subsequent HIV care outcomes. We will examine whether they are mediated by individual-level psychological distress and moderated by coping mechanisms, with maladaptive ones (ie, AOD use) mediating adverse neighborhood effects and positive ones (ie, positive coping skills) buffering them.

A more nuanced understanding of the mechanisms by which neighborhood and network-level factors influence the HIV care continuum steps among MSM will guide the development of urban planning policies and multilevel, neighborhood and network-level interventions to augment individual and patient provider-level interventions to improve outcomes. Neighborhood and network factors are often examined separately in research studies, and this study aims to evaluate both factors using multilevel modeling. Specific interventions can be tailored to subpopulation groups focusing on malleable characteristics that specifically influence HIV care outcomes for that group. For example, high-poverty neighborhoods with numerous health and HIV-related resources that also have high levels of HIV stigma and homophobia may be associated with inconsistent care receipt inside of that neighborhood and, subsequently, low levels of ART adherence for MSM, especially among those with high internalized stigma and social networks that offer less support. In contrast, for care receipt outside the neighborhood, network and individual-level poverty may be more important to care outcomes than local resources or neighborhood stigma. Our planned analyses will identify sets of neighborhood and network-level characteristics that constitute the varying contexts where access to care and engagement are optimized. Results will also identify how individual-level factors interact with these contexts.

Such results could inform the design of care engagement interventions for MSM who receive HIV care inside and outside their neighborhoods. Determining if the association between neighborhood resources and HIV care outcomes is modified by transit access and the neighborhood type (eg, home vs social) could inform future siting of resources for urban MSM living with HIV in underserved areas. Identifying the effects of exposure duration to stressful neighborhoods, through analysis of MSM who move, and network-level influences could inform our understanding of where to site housing for MSM living with HIV and who might benefit from moving to a different neighborhood. Social network–based interventions can be tailored to promote HIV serostatus disclosure, ART adherence, and retention in care within social networks through peer leaders to leverage their influence over HIV care-associated social norms within networks, taking into account how neighborhood-level factors condition network influences. Most importantly, the level of detail and longitudinal nature of the study addresses concerns around one-size-fits-all approaches, even at the neighborhood and network levels, such as generic antistigma media campaigns and crime reduction efforts.

The COVID-19 pandemic caused by the SARS-CoV-2 virus that emerged in New York City in March 2020 was associated with high levels of morbidity and mortality, especially among those who are immunocompromised and have underlying comorbidities, including HIV [106]. Our study had to pause enrollment from March 2020 to August 2021, with the resumption of enrollment in August 2021 to November 2022. This will allow us to conduct additional analysis on the effect of the COVID-19 pandemic on the mental health, substance use, and general well-being of MSM living with HIV in New York City, as well as its effect on how neighborhood and social network factors influence HIV care outcomes pre- and post-pandemic.

### Conclusions

In summary, the study uses an innovative approach combining geospatial mapping of neighborhoods with multilevel modeling, spatial statistics, and an extensive geospatial database. It will have direct implications for the design of multilevel interventions, addressing factors at the neighborhood, network, and individual levels, to improve HIV care outcomes for MSM, particularly for Black and Latino MSM. This study will have direct implications for the design of neighborhood- and network-level interventions and urban planning to improve the HIV care outcomes for MSM, particularly for Black and Latino MSM.

## Appendix

Multimedia Appendix 1 Peer review report from the Population and Public Health Approaches to HIV/AIDS Study Section (PPAH) - AIDS and Related Research Integrated Review Group - Center for Scientific Review (National Institutes of Health, USA).

## Abbreviations

ACTG: AIDS Clinical Trials Group
ADAP: New York State AIDS Drug Assistance Program
AOD: alcohol and other drug
ART: antiretroviral therapy
CASI: computer-assisted survey instrument
CD4 T cell: CD4 T lymphocyte
MSM: men who have sex with men
NNHIV: Neighborhoods, Networks and HIV Care among Men Who Have Sex with Men
SDH: social determinants of health
SNI: social network inventory

## Data Availability

The datasets generated and analyzed during this study are available from the corresponding author on reasonable request.

## References

[1] Maulsby C, Millett G, Lindsey K, Kelley R, Johnson K, Montoya D, Holtgrave D (2014). HIV among black men who have sex with men (MSM) in the United States: a review of the literature. AIDS Behav.

[2] Buchacz K, Baker RK, Palella FJ, Shaw L, Patel P, Lichtenstein KA, Chmiel JS, Vellozzi C, Debes R, Henry K, Overton ET, Bush TJ, Tedaldi E, Carpenter C, Mayer KH, Brooks JT, HIV Outpatient Study Investigators (2013). Disparities in prevalence of key chronic diseases by gender and race/ethnicity among antiretroviral-treated HIV-infected adults in the US. Antivir Ther.

[3] Millett GA, Peterson JL, Flores SA, Hart TA, Jeffries WL, Wilson PA, Rourke SB, Heilig CM, Elford J, Fenton KA, Remis RS (2012). Comparisons of disparities and risks of HIV infection in black and other men who have sex with men in Canada, UK, and USA: a meta-analysis. Lancet.

[4] Beer L, Oster AM, Mattson CL, Skarbinski J, Medical Monitoring Project (2014). Disparities in HIV transmission risk among HIV-infected black and white men who have sex with men, United States, 2009. AIDS.

[5] Hall HI, Frazier EL, Rhodes P, Holtgrave DR, Furlow-Parmley C, Tang T, Gray KM, Cohen SM, Mermin J, Skarbinski J (2013). Differences in human immunodeficiency virus care and treatment among subpopulations in the United States. JAMA Intern Med.

[6] Hightow-Weidman LB, Jones K, Phillips G, Wohl A, Giordano TP, YMSM of Color SPNS Initiative Study Group (2011). Baseline clinical characteristics, antiretroviral therapy use, and viral load suppression among HIV-positive young men of color who have sex with men. AIDS Patient Care STDS.

[7] Axelrad JE, Mimiaga MJ, Grasso C, Mayer KH (2013). Trends in the spectrum of engagement in HIV care and subsequent clinical outcomes among men who have sex with men (MSM) at a Boston Community Health Center. AIDS Patient Care STDS.

[8] Maulsby C, Millett G, Lindsey K, Kelley R, Johnson K, Montoya D, Holtgrave D (2014). HIV among black men who have sex with men (MSM) in the United States: a review of the literature. AIDS Behav.

[9] Hoots BE, Finlayson TJ, Wejnert C, Paz-Bailey G, National HIV Behavioral Surveillance (NHBS) Study Group (2017). Updated data on linkage to human immunodeficiency virus care and antiretroviral treatment among men who have sex with men-20 cities, United States. J Infect Dis.

[10] Beer L, Bradley H, Mattson CL, Johnson CH, Hoots B, Shouse RL, Medical Monitoring Project (2016). Trends in racial and ethnic disparities in antiretroviral therapy prescription and viral suppression in the United States, 2009-2013. J Acquir Immune Defic Syndr.

[11] Gant Z, Bradley H, Hu X, Skarbinski J, Hall H I, Lansky A, Centers for Disease ControlPrevention (CDC) (2014). Hispanics or latinos living with diagnosed HIV: progress along the continuum of HIV care - United States, 2010. MMWR Morb Mortal Wkly Rep.

[12] Crepaz N, Tang T, Marks G, Hall HI (2017). Viral suppression patterns among persons in the United States with diagnosed HIV infection in 2014. Ann Intern Med.

[13] Sprague C, Simon SE (2021). Ending HIV in the USA: integrating social determinants of health. Lancet.

[14] Lua I, Silva AF, Guimarães NS, Magno L, Pescarini J, Anderle RV, Ichihara MY, Barreto ML, Teles Santos CA, Chenciner L, Souza LE, Macinko J, Dourado I, Rasella D (2023). The effects of social determinants of health on acquired immune deficiency syndrome in a low-income population of Brazil: a retrospective cohort study of 28.3 million individuals. Lancet Reg Health Am.

[15] Wohl AR, Galvan FH, Carlos J, Myers HF, Garland W, Witt MD, Cadden J, Operskalski E, Jordan W, George S (2013). A comparison of MSM stigma, HIV stigma and depression in HIV-positive Latino and African American men who have sex with men (MSM). AIDS Behav.

[16] Eberhart MG, Yehia BR, Hillier A, Voytek CD, Fiore DJ, Blank M, Frank I, Metzger DS, Brady KA (2015). Individual and community factors associated with geographic clusters of poor HIV care retention and poor viral suppression. J Acquir Immune Defic Syndr.

[17] Shacham E, Lian M, Önen N F, Donovan M, Overton E (2013). Are neighborhood conditions associated with HIV management?. HIV Med.

[18] Sheehan DM, Cosner C, Fennie KP, Gebrezgi MT, Cyrus E, Maddox LM, Levison JH, Spencer EC, Niyonsenga T, Trepka MJ (2018). Role of country of birth, testing site, and neighborhood characteristics on nonlinkage to HIV care among Latinos. AIDS Patient Care STDS.

[19] Eberhart MG, Yehia BR, Hillier A, Voytek CD, Blank MB, Frank I, Metzger DS, Brady KA (2013). Behind the cascade: analyzing spatial patterns along the HIV care continuum. J Acquir Immune Defic Syndr.

[20] Wohl AR, Galvan FH, Myers HF, Garland W, George S, Witt M, Cadden J, Operskalski E, Jordan W, Carpio F, Lee ML (2011). Do social support, stress, disclosure and stigma influence retention in HIV care for Latino and African American men who have sex with men and women?. AIDS Behav.

[21] Waddell EN, Messeri PA (2006). Social support, disclosure, and use of antiretroviral therapy. AIDS Behav.

[22] Persson L, Ostergren PO, Hanson BS, Lindgren A, Naucler A (2002). Social network, social support and the rate of decline of CD4 lymphocytes in asymptomatic HIV-positive homosexual men. Scand J Public Health.

[23] Goldenberg T, Stephenson R (2015). "The more support you have the better": partner support and dyadic HIV care across the continuum for gay and bisexual men. J Acquir Immune Defic Syndr.

[24] Burgoyne RW (2005). Exploring direction of causation between social support and clinical outcome for HIV-positive adults in the context of highly active antiretroviral therapy. AIDS Care.

[25] Kelly JD, Hartman C, Graham J, Kallen MA, Giordano TP (2014). Social support as a predictor of early diagnosis, linkage, retention, and adherence to HIV care: results from the steps study. J Assoc Nurses AIDS Care.

[26] Pugsley RA, Chapman DA, Kennedy MG, Liu H, Lapane KL (2013). Residential segregation and gonorrhea rates in US metropolitan statistical areas, 2005-2009. Sex Transm Dis.

[27] Biello KB, Kershaw T, Nelson R, Hogben M, Ickovics J, Niccolai L (2012). Racial residential segregation and rates of gonorrhea in the United States, 2003-2007. Am J Public Health.

[28] Adimora AA, Schoenbach VJ, Taylor EM, Khan MR, Schwartz RJ, Miller WC (2013). Sex ratio, poverty, and concurrent partnerships among men and women in the United States: a multilevel analysis. Ann Epidemiol.

[29] Barrera M, Ainlay SL (1983). The structure of social support: a conceptual and empirical analysis. J Community Psychol.

[30] Johnson MO, Neilands TB, Dilworth SE, Morin SF, Remien RH, Chesney MA (2007). The role of self-efficacy in HIV treatment adherence: validation of the HIV treatment adherence self-efficacy scale (HIV-ASES). J Behav Med.

[31] Chesney MA, Ickovics JR, Chambers DB, Gifford AL, Neidig J, Zwickl B, Wu AW (2000). Self-reported adherence to antiretroviral medications among participants in HIV clinical trials: the AACTG adherence instruments. AIDS Care.

[32] Bodenlos JS, Grothe KB, Kendra K, Whitehead D, Copeland AL, Brantley PJ (2004). Attitudes toward HIV health care providers scale: development and validation. AIDS Patient Care STDS.

[33] Chew LD, Bradley KA, Boyko EJ (2004). Brief questions to identify patients with inadequate health literacy. Fam Med.

[34] Carey MP, Morrison-Beedy D, Johnson BT (1997). The HIV-knowledge questionnaire: development and evaluation of a reliable, valid, and practical self-administered questionnaire. AIDS and Behavior.

[35] Willis S, Castel AD, Ahmed T, Olejemeh C, Frison L, Kharfen M (2013). Linkage, engagement, and viral suppression rates among HIV-infected persons receiving care at medical case management programs in Washington, DC. J Acquir Immune Defic Syndr.

[36] Irvine MK, Chamberlin SA, Robbins RS, Myers JE, Braunstein SL, Mitts BJ, Harriman GA, Laraque F, Nash D (2015). Improvements in HIV care engagement and viral load suppression following enrollment in a comprehensive HIV care coordination program. Clin Infect Dis.

[37] Eaton EF, Saag MS, Mugavero M (2014). Engagement in human immunodeficiency virus care: linkage, retention, and antiretroviral therapy adherence. Infect Dis Clin North Am.

[38] Tsuyuki K, Surratt HL (2015). Antiretroviral drug diversion links social vulnerability to poor medication adherence in substance abusing populations. AIDS Behav.

[39] Surratt HL, Kurtz SP, Cicero TJ, O'Grady C, Levi-Minzi MA (2013). Antiretroviral medication diversion among HIV-positive substance abusers in South Florida. Am J Public Health.

[40] Surratt HL, Kurtz SP, Levi-Minzi MA, Chen M (2015). Environmental influences on HIV medication adherence: the role of neighborhood disorder. Am J Public Health.

[41] Bogart LM, Wagner GJ, Green HD, Mutchler MG, Klein DJ, McDavitt B, Lawrence SJ, Hilliard CL (2016). Medical mistrust among social network members may contribute to antiretroviral treatment nonadherence in African Americans living with HIV. Soc Sci Med.

[42] Bogart LM, Thorburn S (2005). Are HIV/AIDS conspiracy beliefs a barrier to HIV prevention among African Americans?. J Acquir Immune Defic Syndr.

[43] Bozzette SA, Berry SH, Duan N, Frankel MR, Leibowitz AA, Lefkowitz D, Emmons CA, Senterfitt JW, Berk ML, Morton SC, Shapiro MF (1998). The care of HIV-infected adults in the United States. HIV cost and services utilization study consortium. N Engl J Med.

[44] Vina ER, Hausmann LRM, Utset TO, Masi CM, Liang KP, Kwoh CK (2015). Perceptions of racism in healthcare among patients with systemic lupus erythematosus: a cross-sectional study. Lupus Sci Med.

[45] LaVeist TA, Nickerson KJ, Bowie JV (2000). Attitudes about racism, medical mistrust, and satisfaction with care among African American and white cardiac patients. Med Care Res Rev.

[46] Galassi JP, Schanberg R, Ware WB (1992). The patient reactions assessment: a brief measure of the quality of the patient-provider medical relationship. Psychol Assess.

[47] Oetzel J, Wilcox B, Avila M, Hill R, Archiopoli A, Ginossar T (2015). Patient-provider interaction, patient satisfaction, and health outcomes: testing explanatory models for people living with HIV/AIDS. AIDS Care.

[48] Humeniuk R, Ali R, Babor TF, Farrell M, Formigoni ML, Jittiwutikarn J, de Lacerda R B, Ling W, Marsden J, Monteiro M, Nhiwatiwa S, Pal H, Poznyak V, Simon S (2008). Validation of the alcohol, smoking and substance involvement screening test (ASSIST). Addiction.

[49] Kroenke K, Spitzer RL, Williams JB (2001). The PHQ-9: validity of a brief depression severity measure. J Gen Intern Med.

[50] Boardman JD, Finch BK, Ellison CG, Williams DR, Jackson JS (2001). Neighborhood disadvantage, stress, and drug use among adults. J Health Soc Behav.

[51] Prins A, Ouimette P, Kimerling R, Camerond RP, Hugelshofer DS, Shaw-Hegwer J, Thrailkill A, Gusman FD, Sheikh JI (2004). The primary care PTSD screen (PC–PTSD): development and operating characteristics. Prim Care Psych.

[52] Landrine H, Klonoff EA (1996). The schedule of racist events: a measure of racial discrimination and a study of its negative physical and mental health consequences. J Black Psychol.

[53] Díaz RM, Ayala G, Bein E, Henne J, Marin BV (2001). The impact of homophobia, poverty, and racism on the mental health of gay and bisexual Latino men: findings from 3 US cities. Am J Public Health.

[54] Ross MW, Rosser BRS (1996). Measurement and correlates of internalized homophobia: a factor analytic study. J Clin Psychol.

[55] Kalichman SC, Simbayi LC, Cloete A, Mthembu PP, Mkhonta RN, Ginindza T (2009). Measuring AIDS stigmas in people living with HIV/AIDS: the internalized AIDS-related stigma scale. AIDS Care.

[56] Earnshaw VA, Smith LR, Chaudoir SR, Amico KR, Copenhaver MM (2013). HIV stigma mechanisms and well-being among PLWH: a test of the HIV stigma framework. AIDS Behav.

[57] Rogers ES, Ralph RO, Salzer MS (2010). Validating the empowerment scale with a multisite sample of consumers of mental health services. Psychiatr Serv.

[58] Wallston KA, Malcarne VL, Flores L, Hansdottir I, Smith CA, Stein MJ, Weisman MH, Clements PJ (1999). Does god determine your health? The god locus of health control scale. Cogn Behav Ther.

[59] Arciniega GM, Anderson TC, Tovar-Blank ZG, Tracey TJG (2008). Toward a fuller conception of machismo: development of a traditional machismo and caballerismo scale. J Couns Psychol.

[60] Galvan FH, Bogart LM, Wagner GJ, Klein DJ, Chen YT (2014). Conceptualisations of masculinity and self-reported medication adherence among HIV-positive Latino men in Los Angeles, California, USA. Cult Health Sex.

[61] Carver CS (1997). You want to measure coping but your protocol's too long: consider the brief COPE. Int J Behav Med.

[62] Grov C (2012). HIV risk and substance use in men who have sex with men surveyed in bathhouses, bars/clubs, and on craigslist.org: venue of recruitment matters. AIDS Behav.

[63] Frost DM, Meyer IH (2009). Internalized homophobia and relationship quality among lesbians, gay men, and bisexuals. J Couns Psychol.

[64] Marin G, Sabogal F, Marin BV, Otero-Sabogal R, Perez-Stable EJ (2016). Development of a short acculturation scale for hispanics. Hisp J Behav Sci.

[65] Cerdá M, Nandi V, Frye V, Egan JE, Rundle A, Quinn JW, Sheehan D, Hoover DR, Ompad DC, van Tieu H, Greene E, Koblin B (2017). Neighborhood determinants of mood and anxiety disorders among men who have sex with men in New York City. Soc Psychiatry Psychiatr Epidemiol.

[66] Frye V, Nandi V, Egan J, Cerda M, Greene E, van Tieu H, Ompad DC, Hoover DR, Lucy D, Baez E, Koblin BA (2015). Sexual orientation- and race-based discrimination and sexual HIV risk behavior among urban MSM. AIDS Behav.

[67] Koblin BA, Egan JE, Rundle A, Quinn J, Tieu H, Cerdá M, Ompad DC, Greene E, Hoover DR, Frye V (2013). Methods to measure the impact of home, social, and sexual neighborhoods of urban gay, bisexual, and other men who have sex with men. PLoS One.

[68] Sampson RJ, Morenhoff J, Gannon-Rowley T (2002). Assessing ‘Neighborhood Effects’: social processes and new directions in research. Annu Rev Sociol.

[69] Sampson RJ (2003). The neighborhood context of well-being. Perspect Biol Med.

[70] Sampson RJ, Groves WB (1989). Community structure and crime: testing social-disorganization theory. Am J Sociol.

[71] Browning CR, Cagney KA (2002). Neighborhood structural disadvantage, collective efficacy, and self-rated physical health in an urban setting. J Health Soc Behav.

[72] Sampson RJ, Raudenbush SW, Earls F (1997). Neighborhoods and violent crime: a multilevel study of collective efficacy. Science.

[73] Sampson RJ, Morenoff JD, Brooks-Gunn J, Duncan GJ, Aber JL (1997). Ecological Perspectives on the Neighborhood Context of Urban Poverty: Past and Present, in Neighborhood Poverty: Vol 2 Policy Implications in Studying Neighborhoods.

[74] Ross CE, Mirowsky J (1999). Disorder and decay: the concept and measurement of perceived neighborhood disorder. Urban Aff Rev.

[75] Hastings TL, Kelley ML (1997). Development and validation of the screen for adolescent violence exposure (SAVE). J Abnorm Child Psychol.

[76] Perkins DD, Florin P, Rich RC, Wandersman A, Chavis DM (1990). Participation and the social and physical environment of residential blocks: crime and community context. American J of Comm Psychol.

[77] Cameron JE (2004). A three-factor model of social identity. Self Identity.

[78] Latkin CA, Yang C, Tobin KE, German D (2013). Injection drug users' and their risk networks' experiences of and attitudes towards drug dealer violence in Baltimore, Maryland. Int J Drug Policy.

[79] Wright D (2002). State Estimates of Substance Use From the 2000 National Household Survey on Drug Abuse: Volume I. Findings.

[80] Krieger N (1990). Racial and gender discrimination: risk factors for high blood pressure?. Soc Sci Med.

[81] Park Y, Quinn J, Florez K, Jacobson J, Neckerman K, Rundle A (2011). Hispanic immigrant women's perspective on healthy foods and the New York City retail food environment: a mixed-method study. Soc Sci Med.

[82] Park Y, Neckerman KM, Quinn J, Weiss C, Rundle A (2008). Place of birth, duration of residence, neighborhood immigrant composition and body mass index in New York City. Int J Behav Nutr Phys Act.

[83] Park Y, Neckerman K, Quinn J, Weiss C, Jacobson J, Rundle A (2011). Neighbourhood immigrant acculturation and diet among Hispanic female residents of New York City. Public Health Nutr.

[84] Amirkhanian YA, Kelly JA, Takacs J, Kuznetsova AV, DiFranceisco WJ, Mocsonaki L, McAuliffe TL, Khoursine RA, Toth TP (2009). HIV/STD prevalence, risk behavior, and substance use patterns and predictors in Russian and Hungarian sociocentric social networks of men who have sex with men. AIDS Educ Prev.

[85] Walls DW (2007). National establishment time-series database©: data overview. SSRN.

[86] Sullivan PS, Woodyatt C, Koski C, Pembleton E, McGuinness P, Taussig J, Ricca A, Luisi N, Mokotoff E, Benbow N, Castel AD, Do AN, Valdiserri RO, Bradley H, Jaggi C, O'Farrell D, Filipowicz R, Siegler AJ, Curran J, Sanchez TH (2020). A data visualization and dissemination resource to support HIV prevention and care at the local level: analysis and uses of the AIDSVu public data resource. J Med Internet Res.

[87] Mahajan A, Sayles JN, Patel VA, Remien RH, Sawires SR, Ortiz DJ, Szekeres G, Coates TJ (2008). Stigma in the HIV/AIDS epidemic: a review of the literature and recommendations for the way forward. AIDS.

[88] Logie C, Gadalla TM (2009). Meta-analysis of health and demographic correlates of stigma towards people living with HIV. AIDS Care.

[89] Golub SA, Gamarel KE (2013). The impact of anticipated HIV stigma on delays in HIV testing behaviors: findings from a community-based sample of men who have sex with men and transgender women in New York City. AIDS Patient Care STDS.

[90] Mannheimer SB, Wang L, Wilton L, van Tieu H, Del Rio C, Buchbinder S, Fields S, Glick S, Connor MB, Cummings V, Eshleman SH, Koblin B, Mayer KH (2014). Infrequent HIV testing and late HIV diagnosis are common among a cohort of black men who have sex with men in 6 US cities. J Acquir Immune Defic Syndr.

[91] Oldenburg CE, Perez-Brumer AG, Hatzenbuehler ML, Krakower D, Novak DS, Mimiaga MJ, Mayer KH (2015). State-level structural sexual stigma and HIV prevention in a national online sample of HIV-uninfected MSM in the United States. AIDS.

[92] Eaton LA, Driffin DD, Kegler C, Smith H, Conway-Washington C, White D, Cherry C (2015). The role of stigma and medical mistrust in the routine health care engagement of black men who have sex with men. Am J Public Health.

[93] Biello KB, Oldenburg CE, Mitty JA, Closson EF, Mayer KH, Safren SA, Mimiaga MJ (2017). The "Safe Sex" conundrum: anticipated stigma from sexual partners as a barrier to PrEP use among substance using MSM engaging in transactional sex. AIDS Behav.

[94] Golub SA, Gamarel KE, Rendina HJ, Surace A, Lelutiu-Weinberger CL (2013). From efficacy to effectiveness: facilitators and barriers to PrEP acceptability and motivations for adherence among MSM and transgender women in New York City. AIDS Patient Care STDS.

[95] Farhat D, Greene E, Paige MQ, Koblin BA, Frye V (2017). Knowledge, stereotyped beliefs and attitudes around HIV chemoprophylaxis in two high HIV prevalence neighborhoods in New York City. AIDS Behav.

[96] Folkman S (20211). The Oxford Handbook of Stress, Health, and Coping.

[97] Meyer IH (2003). Prejudice, social stress, and mental health in lesbian, gay, and bisexual populations: conceptual issues and research evidence. Psychol Bull.

[98] Diez Roux AV, Mair C (2010). Neighborhoods and health. Ann N Y Acad Sci.

[99] Ross CE, Mirowsky J (2001). Neighborhood disadvantage, disorder, and health. J Health Soc Behav.

[100] O’Brien DT, Sampson RJ (2015). Public and private spheres of neighborhood disorder. J Res Crime Delinq.

[101] Kirst M, Lazgare LP, Zhang YJ, O'Campo P (2015). The effects of social capital and neighborhood characteristics on intimate partner violence: a consideration of social resources and risks. Am J Community Psychol.

[102] Shmool JLC, Yonas MA, Newman OD, Kubzansky LD, Joseph E, Parks A, Callaway C, Chubb LG, Shepard P, Clougherty JE (2015). Identifying perceived neighborhood stressors across diverse communities in New York City. Am J Community Psychol.

[103] Voisin DR, Jenkins EJ, Takahashi L (2011). Toward a conceptual model linking community violence exposure to HIV-related risk behaviors among adolescents: directions for research. J Adolesc Health.

[104] Javdani S, Abdul-Adil J, Suarez L, Nichols SR, Farmer AD (2014). Gender differences in the effects of community violence on mental health outcomes in a sample of low-income youth receiving psychiatric care. Am J Community Psychol.

[105] Copeland-Linder N, Lambert SF, Chen YF, Ialongo NS (2011). Contextual stress and health risk behaviors among African American adolescents. J Youth Adolesc.

[106] Braunstein SL, Wahnich A, Lazar R (2023). COVID-19 outcomes among people with HIV and COVID-19 in New York City. J Infect Dis.

