# Supplementary material for: Neighborhoods, Networks, and HIV Care Among Men Who Have Sex With Men: Proposal for a Longitudinal Study
Source: JMIR Res Protoc. 2024 Nov 13;13:e64358. doi: 10.2196/64358 (PMC11602767; doi:10.2196/64358)
Supplement: Multimedia Appendix 1 [file resprot_v13i1e64358_app1.pdf]

1R01MH118960-01A1 Tieu, Hong Van

EARLY STAGE INVESTIGATOR
NEW INVESTIGATOR

RESUME AND SUMMARY OF DISCUSSION: This application seeks to identify neighborhood and network characteristics that account for racial/ethnic disparities along the HIV care continuum for Latino and Black MSM living with HIV/AIDS in New York City. The premise of this application is strong as Black and Latino MSM are more likely to have lower rates than white or other ethnic groups of PrEP uptake; retention in care, and viral suppression. The PI and his collaborators are very well qualified to carry out this research having demonstrated experience in similar projects and a solid track record of collaboration. The applicants intend to conduct spatial analysis to characterize neighborhoods and networks, assessing longitudinal changes in both, and identifying how these are related to HIV retention in care and adherence. This is a rather innovative approach that promises to inform future interventions. The application is conceptually grounded and the hypotheses are clearly delineated. This resubmission is very well written and is very responsive to prior critiques. Among the many improvements implemented are the following: the applicants have addressed the generalizability of the study even if it is carried out in NYC; they have expanded their eligibility criteria to include Latino/Hispanic MSM and have grounded this research in appropriate theories. Some minor weaknesses were raised about the lack of clarity around the mediators and moderators in the modeling, and the selection bias that might ensue from the challenges in transportation some of these MSM may face in commuting to Manhattan for their initial interview. Despite these, the committee was confident this application would significantly impact the development of interventions sensitive to structural contexts. As such, the overwhelming majority of reviewers assessed its potential overall impact as high.

DESCRIPTION (provided by applicant): Marked disparities by race/ethnicity exist in HIV infection rates and engagement and retention in care among men who have sex with men (MSM) along the HIV care continuum. Compared with White MSM, HIV- infected Black and Hispanic/Latino MSM are less likely to be on antiretroviral therapy (ART), adhere to ART, and achieve viral suppression. Lower rates of care engagement and retention among Black and Latino MSM are influenced by factors beyond the individual. In order to uncover the neighborhood- and network-involved pathways that produce racial/ethnic disparities in HIV care outcomes, yielding an empirical basis for interventions and policies to fill gaps in the care continuum among Black and Latino MSM, systematic, theory- based investigation of the specific and intersecting neighborhood and network characteristics that relate to retention in care, ART adherence, and viral suppression must be engaged. Using socioecological and intersectional conceptual frameworks, several mid-range and micro social and psychological theories, and informed by our NIH/NIMH R56-funded formative research, we propose to identify neighborhood-and network-level characteristics associated with HIV care continuum outcomes (viral suppression, retention in care, and ART adherence) among 550 HIV-infected MSM living in New York City (NYC) via a longitudinal study. We will investigate the influence of neighborhood composition and four neighborhood-level characteristics domains, testing theoretical pathways of influence across the different neighborhoods in which MSM live, socialize, and receive HIV care. The Specific Aims are, among HIV-infected MSM in NYC: Aim 1: To use spatial analysis to characterize clustering and spatial trends in HIV care outcomes. Aim 2: To examine HIV care outcomes by race/ethnicity in relationship to longitudinal changes in: 2a) Exposure to neighborhoods of potential influence (home; social; health facility access); 2b) Neighborhood characteristics conceptual domains (community violence/physical disorder/social disorganization; AOD use factors; social norms; community resources); 2c) Social networks. Aim 3: To use multilevel modeling to assess associations among changes over time in exposure to the neighborhood characteristics and changes in social networks with HIV care outcomes by race/ethnicity, while assessing the potential mediating and moderating effects of individual- and network-level factors. The study will employ an innovative approach combining geospatial mapping of neighborhoods with multilevel modeling, spatial statistics, and an existing, extensive geospatial database. It will have direct

implications for the design of multilevel interventions, addressing factors at the neighborhood-, network-, and individual-levels, to improve HIV care outcomes for MSM, particularly for Black and Latino MSM.

PUBLIC HEALTH RELEVANCE: The study purpose is to examine the neighborhoods in which HIV-infected men who have sex with men (MSM) in New York City live, socialize, and get HIV medical care and the structure and make-up of social networks (connections between friends, family members, etc.) over time, and to assess the relationship of these characteristics to achieving undetectable HIV viral load, being retained in HIV care, and adhering to HIV drugs. The study will also examine how these factors differ by race/ethnicity. This study will have direct impact on the design of programs to improve HIV care among MSM, particularly for Black and Hispanic/Latino MSM.

CRITIQUE 1

Significance: 1
Investigator(s): 2
Innovation: 1
Approach: 4
Environment: 1

Overall Impact: The proposed resubmission study aims to address ethnic/racial disparities in retention in HIV care, ART adherence, and viral load among HIV-infected MSM in NYC by exploring multi-level predictors of the HIV care continuum: neighborhood, social network, and individual-level variables, and thus, has a strong scientific premise. The environment to conduct this research is excellent and is led by an experienced team with a history of collaborating. The PI, Dr. Tieu is an ESI and NI. The use of spatial analysis in the context of network analysis to address disparities in the HIV continuum is highly innovative. The approach includes several strengths, including a biological primary outcome (viral load), spatial analysis, a large proposed sample, and a history of success recruiting the target population. There are also several moderate methodological weaknesses, chiefly lack of clarity in modeling longitudinal mediation, and lack of specificity of proposed mediators/moderators. If the aims of the study are accomplished, it is likely to have a strong impact on the field.

1. Significance:

Strengths

- Addresses key areas in the HIV care continuum—retention in care, ART adherence, and viral suppression
- Focuses on MSM, with oversampling of MSM of color—a highly vulnerable group in which HIV care disparities are salient
- Integrates multi-level predictors of the HIV care continuum—neighborhood, social network, and individual-level constructs

Weaknesses

- None Noted

2. Investigator(s):

Strengths

- PI and Co-Is have established records of conducting research on HIV risk and care among MSM
- Study team has a history of collaborating

Weaknesses

- No mental health expert on study team

3. Innovation:

Strengths

- The application of neighborhood and network analysis to HIV care has yet to be explored fully in context of each other
- Use of spatial analysis addresses past limitations in neighborhood-level research in this area

Weaknesses

- None Noted

4. Approach:

Strengths

- Objective biological outcome of viral load suppression is included
- Pilot data from study team shows promise in recruitment/retention of the proposed sample
- Application of spatial analysis in conjunction with network analysis strengthens the methodology of the application
- Large sample size planned, with a large proportion of MSM of color, which reduces error and increases generalizability

Weaknesses

- No exclusion criteria listed; no upper age limit stated
- Adherence operationally defined at 95+%, but no justification is provided. It is also unclear if such a high threshold is optimal for classifying adherence, given newer ART typically confers clinical benefit at 80+%
- It’s unclear how self-report and pill count/pharmacy data will be reconciled if inconsistencies emerge
- Unclear what gender role measure will be used for non-Latino participants
- Mediational analyses are planned with the baseline data; mediation based on cross-sectional designs are significantly biased and do not provide meaningful information on mediational process, which by definition occur temporally.
- Insufficient information is provided for the plan to test longitudinal mediation. State of the science in this area includes the use of sequential processing with latent growth curve models and latent difference (or latent change) scores. It is not clear how the statistical analysis plan addresses a crucial requirement for mediation: the temporal ordering of independent, mediator, and dependent variables
- It is not clear what measures are planned for analysis as mediators and moderators

5. Environment:

Strengths

- Excellent environment with more than adequate resources to accomplish the stated aims of the project

Weaknesses

- None Noted

Protections for Human Subjects:

Acceptable Risks and/or Adequate Protections

- Generally acceptable, although the researchers may wish to have procedures in place for individuals who report elevated levels of depression, anxiety, or substance use. Alternately, referral information for mental health/substance use treatment could be provided to all participants at baseline.

Data and Safety Monitoring Plan (Applicable for Clinical Trials Only):

Not Applicable (No Clinical Trials)

Inclusion of Women, Minorities and Children:

- Sex/Gender: Distribution justified scientifically
- Race/Ethnicity: Distribution justified scientifically
- For NIH-Defined Phase III trials, Plans for valid design and analysis: Not applicable
- Inclusion/Exclusion of Children under 18: Including ages <18; justified scientifically
- Acceptable and well justified

Vertebrate Animals:

Not Applicable (No Vertebrate Animals)

Biohazards:

Acceptable

- Accentuable plan for sampling of viral load and CD4

Resubmission:

- The resubmission has generally been responsive to the initial set of critiques, although mediation analyses have not been fully clarified

Resource Sharing Plans:

Acceptable

- acceptable plan for sharing

Authentication of Key Biological and/or Chemical Resources:

- NA

Budget and Period of Support:

Recommend as Requested:

CRITIQUE 2

Significance: 2
Investigator(s): 3
Innovation: 3
Approach: 3
Environment: 2

Overall Impact: This is a revised R01 project with a goal to study the HIV care continuum for HIV-infected MSM in NYC, which is proposed by a new investigator, Dr. Tieu, Associate Member at the Lab of Infectious Disease Prevention, New York Blood Center. The PI has good training in medicine (MD) and epidemiology (MS), which is appropriate for the proposed project. The PI has accumulated experience for the proposed project from other similar studies. Other team members are also qualified for the proposed tasks. Professional statisticians are included in the study team for data analysis. The proposed aims are important to control HIV infection among MSM populations although it may not be generalizable to other cities under different environments. The study design is well described and appropriate. The preliminary data are provided. The data analyses for each aim are well planned. The research environment at the performance sites are excellent and the institutional support is strong.

1. Significance:

Strengths

- The proposed aims to study the HIV care continuum among HIV-infected MSM in NYC are important and significant.
- If the proposed aims are achieved, it may help to control HIV infection among MSM

Weaknesses

- The proposed study and results may not be generalizable to other cities under different environments.

2. Investigator(s):

Strengths

- The PI, Dr. Tieu, is trained in medicine (MD) and epidemiology (MS) in infectious diseases with appropriate experience and track record for the proposed project.
- The PI has a good publication record on the relevant research topic.
- Other team members are well qualified to perform the proposed role and tasks.
- Statisticians are appropriately included on the team to conduct the data analysis.
- The team members have prior experience collaborating.

Weaknesses

- None Noted

3. Innovation:

Strengths

- It is novel to use neighborhood and network data in the context of longitudinal changes to study the HIV care continuum among HIV-infected MSM.
- Multiple neighborhood contexts are considered.
- It is innovative to use Google Earth and spatial analysis to study the HIV care outcome.

Weaknesses

- The data analysis methods are quite standard although sophisticated.

4. Approach:

Strengths

- The study design is well described and appropriate.
- Preliminary results based on an ongoing study are provided.
- The data analyses for each aim are well planned.

Weaknesses

- None Noted

5. Environment:

Strengths

- The research environment at the performance sites is excellent.
- The institutional support is strong.

Weaknesses

- None Noted

Protections for Human Subjects:

Acceptable Risks and/or Adequate Protections

Data and Safety Monitoring Plan (Applicable for Clinical Trials Only):

Acceptable

Inclusion of Women, Minorities and Children:

- Sex/Gender: Distribution justified scientifically
- Race/Ethnicity: Distribution justified scientifically
- For NIH-Defined Phase III trials, Plans for valid design and analysis: Not applicable
- Inclusion/Exclusion of Children under 18: Including ages <18; justified scientifically

Vertebrate Animals:

Not Applicable (No Vertebrate Animals)

Biohazards:

Not Applicable (No Biohazards)

Resubmission:

- The application was very responsive to prior critiques

Resource Sharing Plans:

Acceptable

Authentication of Key Biological and/or Chemical Resources:

Not Applicable (No Relevant Resources)

Budget and Period of Support:

Recommend as Requested:

CRITIQUE 3

Significance: 2
Investigator(s): 1
Innovation: 2
Approach: 2
Environment: 1

Overall Impact: The proposed project has a strong research premise given the existing race/ethnicity disparities in HIV infection rates, need for resource networks that can achieve greater engagement rates to ultimately improve HIV care outcomes, and prior research that supports the proposed hypothesis. The approach has some very strong aspects including characterization of neighborhood and network characteristics that influence HIV care, the use of multilevel models to measure changes over time across several sociodemographic characteristics, and an overall well-designed longitudinal study that assesses multiple HIV care outcomes. Most of the weaknesses were minor. One minor weakness was the lack of discussion about the ability for the study participants to travel to their research site in Manhattan for the initial interview. Despite the minor weaknesses, the potential overall impact of this project remains high because it will provide critical information on the role of neighborhood and network-level characteristics that lead to racial/ethnic disparities in HIV care outcomes and subsequently inform design of HIV intervention strategies.

1. Significance:

Strengths

- Understanding racial/ethnic disparities in HIV care is an important public health goal.

- Previous studies by the investigators support the proposed hypotheses.
- If successful, the project results will inform HIV intervention strategies in HIV-vulnerable populations.

Weaknesses

- None Noted

2. Investigator(s):

Strengths

- The PI is a new investigator with expertise in HIV epidemiology and network-based studies for MSM.
- The PI has assembled a team of investigators with complementary expertise in social sciences, statistics, and epidemiology to successfully achieve the proposed study.

Weaknesses

- None Noted

3. Innovation:

Strengths

- The characterization of multiple neighborhood contexts to assess their role on HIV care outcomes.
- The characterization of neighborhoods and networks over time to measure changes is innovative.

Weaknesses

- Many of the proposed approaches are standard.
- There have been many studies on racial/ethnic disparities in HIV care.

4. Approach:

Strengths

- Characterization of neighborhood and network characteristics that influence HIV care
- The use of multilevel models to measure changes over time across several sociodemographic characteristics and multiple neighborhoods.
- A well-designed longitudinal study that assesses multiple HIV care outcomes (e.g., viral suppression, retention in care, ART adherence).

Weaknesses

- Given that the population under study may have limited resources, it is not clear if study participants will be able to travel to the research site in Manhattan for the initial interview.
- It is not clear how the study results will be able to inform design of care engagement interventions.

5. Environment:

Strengths

- Excellent research environment to conduct the proposed research.
- Strong letters of support from all participating institutions.

Weaknesses

- None Noted

Protections for Human Subjects:

Acceptable Risks and/or Adequate Protections

- MSM who are HIV positive and over 16 years of age

Data and Safety Monitoring Plan (Applicable for Clinical Trials Only):

Acceptable

Inclusion of Women, Minorities and Children:

- Sex/Gender: Distribution justified scientifically
- Race/Ethnicity: Distribution justified scientifically
- For NIH-Defined Phase III trials, Plans for valid design and analysis:
- Inclusion/Exclusion of Children under 18: Including ages <18; justified scientifically

Vertebrate Animals:

Not Applicable (No Vertebrate Animals)

Biohazards:

Not Applicable (No Biohazards)

Resource Sharing Plans:

Acceptable

Authentication of Key Biological and/or Chemical Resources:

Not Applicable (No Relevant Resources)

Budget and Period of Support:

Recommend as Requested:

THE FOLLOWING SECTIONS WERE PREPARED BY THE SCIENTIFIC REVIEW OFFICER TO SUMMARIZE THE OUTCOME OF DISCUSSIONS OF THE REVIEW COMMITTEE, OR REVIEWERS' WRITTEN CRITIQUES, ON THE FOLLOWING ISSUES:

PROTECTION OF HUMAN SUBJECTS: ACCEPTABLE

INCLUSION OF WOMEN PLAN (G3A): ACCEPTABLE

INCLUSION OF MINORITIES PLAN (M1A): ACCEPTABLE

INCLUSION OF CHILDREN PLAN (C1A): ACCEPTABLE

COMMITTEE BUDGET RECOMMENDATIONS: The budget was recommended as requested.

Footnotes for 1 R01 MH118960-01A1; PI Name: Tieu, Hong Van Nhu

NIH has modified its policy regarding the receipt of resubmissions (amended applications). See Guide Notice NOT-OD-14-074 at http://grants.nih.gov/grants/guide/notice-files/NOT-OD-14-074.html. The impact/priority score is calculated after discussion of an application by averaging the overall scores (1-9) given by all voting reviewers on the committee and multiplying by 10. The criterion scores are submitted prior to the meeting by the individual reviewers assigned to an application, and are not discussed specifically at the review meeting or calculated into the overall impact score. Some applications also receive a percentile ranking. For details on the review process, see http://grants.nih.gov/grants/peer_review_process.htm#scoring.
